# Contrasting effects of visiting urban green-space and the countryside on biodiversity knowledge and conservation support

**DOI:** 10.1371/journal.pone.0174376

**Published:** 2017-03-23

**Authors:** Deborah F. Coldwell, Karl L. Evans

**Affiliations:** Animal and Plant Sciences, Alfred Denny Building, University of Sheffield, Western Bank, Sheffield, United Kingdom; University of Sydney, AUSTRALIA

## Abstract

Conservation policy frequently assumes that increasing people’s exposure to green-space enhances their knowledge of the natural world and desire to protect it. Urban development is, however, considered to be driving declining connectedness to nature. Despite this the evidence base supporting the assumption that visiting green-spaces promotes biodiversity knowledge and conservation support, and the impacts of urbanization on these relationships, is surprisingly limited. Using data from door-to-door surveys of nearly 300 residents in three pairs of small and large urban areas in England we demonstrate that people who visit green-space more regularly have higher biodiversity knowledge and support for conservation (measured using scales of pro-environmental behavior). Crucially these relationships only arise when considering visits to the countryside and not the frequency of visits to urban green-space. These patterns are robust to a suite of confounding variables including nature orientated motivations for visiting green-space, socio-economic and demographic factors, garden-use and engagement with natural history programs. Despite this the correlations that we uncover cannot unambiguously demonstrate that visiting the countryside improves biodiversity knowledge and conservation support. We consider it likely, however, that two mechanisms operate through a positive feedback loop i.e. increased visits to green-space promote an interest in and knowledge of biodiversity and support for conservation, which in turn further increase the desire to visit green-space and experience nature. The intensity of urbanization around peoples’ homes, but not city size, is negatively associated with their frequency of countryside visits and biodiversity knowledge. Designing less intensely urbanized cities with good access to the countryside, combined with conservation policies that promote access to the countryside thus seems likely to maximize urban residents’ biodiversity knowledge and support for conservation.

## Introduction

Conservation biologists have long assumed that engagement with the natural world increases understanding and knowledge of biodiversity, and thus willingness to protect it [1, 2]. Yet, this engagement, for example visiting green-spaces or nature based recreation, is declining due to socio-economic trends including increasing urbanization [2–4]. The conservation community is concerned that this reduced engagement with nature will lead to declines in biodiversity knowledge and conservation support [5, 6]. Indeed, the ‘pigeon paradox’ [7] states that ultimately urbanization will result in support for global conservation relying mainly on peoples’ connection with a small number of common urban species, such as the feral pigeon *Columba livia*, that are of limited conservation concern.

Widespread belief in the hypotheses that engagement with the natural world increases biodiversity knowledge and conservation support has driven investment in numerous conservation initiatives and policies that aim to reconnect people, particularly city dwellers, with nature at local, regional and national scales [8, 9]. The pervasive impact of these hypotheses on conservation policy contrasts somewhat with the quality and quantity of their evidence base. Biodiversity and environmental knowledge amongst the general public appears to be limited [10, 11]. This may partly be an artefact of using hard scientific definitions of knowledge, resulting in poorer knowledge scores than would be obtained through testing the ability of participants to form more abstract mental constructs of biodiversity [12, 13]. Moreover, the relationship between biodiversity knowledge and engagement with nature, through activities such as visiting green-space, has rarely been quantified and is poorly understood [14]. One difficulty in understanding this relationship, in addition to the paucity of studies, is that much of the research that assess biodiversity knowledge recruits participants in green-spaces, and thus do not adequately sample people with very low or zero visitation rates to green-space (e.g. [10,15,16]). Furthermore, people with more knowledge, such as dedicated naturalists, may be motivated to visit green-spaces frequently which hinders disentangling cause and effect. A major complication for research assessing predictors of conservation support is that environmental attitudes are frequently poor predictors of actual behavior [17,18]. Studies that assess how nature engagement influences pro-environmental behaviors and conservation support are also infrequent. They typically compare people that regularly engage in outdoor recreational activity with those who do not [19], and whilst valuable they focus only on the extremes and thus cannot fully quantify the relationship between engagement and conservation support. Other research assessing whether engagement with nature is positively associated with environmental attitudes often use methods that rely on potentially unreliable long-term memories of respondents working in ecology or conservation that have an inherent self-interest in the study [17].

The impacts of urbanization on engagement with nature, ecological knowledge and conservation support are rarely explicitly quantified, and those studies that do so provide conflicting evidence [5]. Initial research typically concluded that environmental support was greater amongst urban than rural residents. This was attributed to urban dwellers’ higher educational standards, greater access to pro-environmental facilities (e.g. recycling), and experience of more degraded environments than people who live in the countryside [20]. In contrast, more recent studies have found fewer differences in environmental attitudes between urban and rural populations [21, 22]. The reasons for this shift are unclear, but may include real temporal shifts in attitudes and behaviors or methodological artefacts, such as more recent analyses taking greater care to control for confounding socio-demographic variables. Whilst urbanization appears to reduce visits to green-space [23] the apparent similarity in environmental attitudes of people living in urban and rural areas contrasts notably with the hypothesis that increasing urbanization reduces engagement with nature and thus support for conservation.

**Fig 1:**
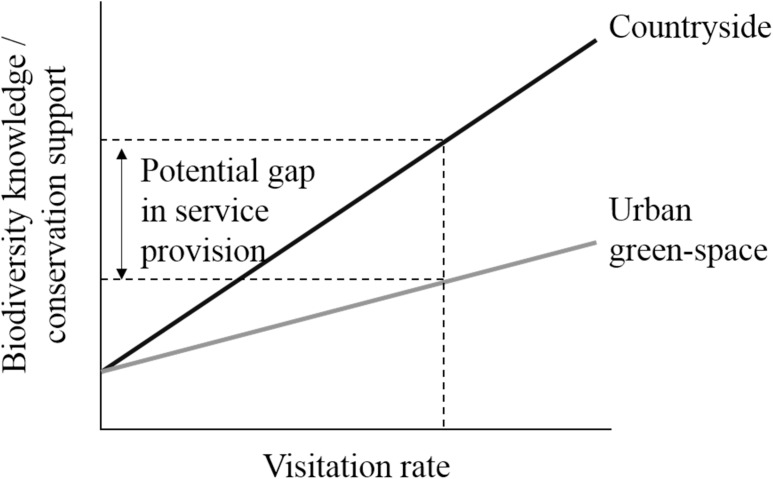
Potential ecosystem service provision gap between urban and rural green-spaces. The slopes of the relationships between green-space visitation rate and biodiversity knowledge (i.e. educational ecosystem services) and associated support for conservation may be shallower for visits to urban than rural green-space, generating a gap in provision of educational ecosystem services between urban and rural green-space. This divergence in service provision may arise from exposure to less diverse biotic assemblages in urban areas that typically comprise generalist species of limited conservation concern.

One crucial aspect of how urbanization influences engagement with nature which has received surprisingly little attention is whether visits to urban green-space can compensate for reduced frequency of visits to the countryside. Green-space in highly developed urban environments typically supports fewer and less specialized species than more natural environments, even in regions where the countryside is dominated by ecologically damaging intensive agriculture [24]. Exposure to biodiversity is assumed to play a causal role in generating biodiversity knowledge and conservation support [2]. Thus a given visit rate to urban green-space may contribute less to knowledge and support than an equivalent visit rate to more natural green-space creating a gap in the provision of educational ecosystem services, i.e. biodiversity knowledge, and associated conservation support (Fig 1).

Despite recent improvements in understanding there is a need to develop the evidence base for a central assumption of conservation theory and policy, i.e. engaging with nature by visiting green-spaces increases biodiversity knowledge and conservation support. The influence of urbanization on these relationships is also insufficiently clear. Here, we use interview data from nearly 300 urban dwellers that were selected in an unbiased manner regarding their use of green-spaces and their socio-economic profile from three pairs of large and small urban areas in England. We test the hypotheses that i) there are positive associations between green-space visitation rates, biodiversity knowledge and conservation support, and ii) that the form of these relationships varies depending on whether visits are to urban green-space or the countryside. We then test the hypotheses that green-space visitation rates, biodiversity knowledge and conservation support are negatively associated with iii) the magnitude of urbanization around people’s homes and iv) the size of urban areas.

## Methods

### Survey sites and urbanization metrics

Surveys were conducted from July to early October 2013 in three pairs of large and small urban areas across England (Manchester and Blackburn; Leeds and Harrogate; Bristol and Bath). Large urban areas comprised between 32 and 122km^2^ of urban land, defined as 1km grid cells with >25% coverage of impervious surface, small urban areas had a spatial extent of 10–18km^2^. To ensure a clear difference between small and large urban areas the former were at least three times smaller than their paired larger urban area. Whilst we do not include mega-cities our focal urban areas are similar in spatial extent to numerous European cities. Paired urban areas were separated by at least 12km of countryside, ensuring that they were distinct (i.e. not part of the same conurbation) and that residents had similar access to countryside. City size is our first urbanization metric.

Our second urbanization metric is the intensity of local scale urbanization surrounding the home of each respondent. Any definition of the distance that is close to a respondent’s home will be somewhat arbitrary but we quantify local scale urbanization within a 1km grid cell centered on each survey post-code. This equates to a radius of 500m from a respondent’s home, matching both the definitions used in a number of other studies as well as policy guidance on accessibility of green-space within 500m of a household [25–27]. The 500m definition represents a radius of c. 5 minutes’ walk from the respondent’s home postcode, based on a direct access route and the average walking speed of 5kmhr^-1^ [28]. Scores are calculated using image recognition software to generate a single urbanization metric based on a semi-automated assessment of the occurrence and density of buildings, roads, other impervious surface, and vegetated green-space from google earth aerial photographs (S1 Fig) within 100 equally sized cells within the focal 1km grid cell. Specifically, the urbanisation index [29] is calculated using a principal component analysis (PCA) of the number of cells with high building density (>50% cover), number of cells with high vegetation density (>50% cover), number of cells containing roads, mean building density score and mean vegetation density score. These scores were used as a measure of how intensively urbanized the area around respondents’ homes were, with a higher score indicative of a more urbanized location. Ground truthing confirmed that images accurately represented land cover at the time of the door-to-door surveys.

### Participant recruitment and sample size

All aspects of the study, including consent procedures, were given ethical approval by the University of Sheffield’s Research Ethics Committee. Informed oral consent was obtained from all participants by asking if they were willing to take part once the rationale and terms of involvement of the study had been explained, which included stating that participants were under no obligation to answer all questions and could stop the survey at any point. We used verbal rather than written consent in part due to the lengthy nature of the questionnaire, and the additional time taken to obtain written consent, and due to concerns of respondents being suspicious of signing contract like documents. If informed consent was not provided no data were collected. Participants were asked again on completion of the survey if they consented to all their answers being used, and if not which answers should not be used.

Door-to-door interviews were conducted during weekday and weekend afternoons and evenings. 20 to 25 postcodes (with at least 15 households) were randomly selected within a 3 km radius of the geographic center of each urban area. All households were approached to take part in a face-to-face interview. Second, the Index of Multiple Deprivation [30] of each respondents’ lower super output area (the smallest spatial unit used in the National Census) was used as an indicator of respondents’ socio-economic profile. The distribution of these profiles was compared to that of the urban area to check that survey respondents were representative, and additional postcodes were randomly selected if deprivation indices were under-represented in the original sample. We obtained 286 completed questionnaires (S1 Table), with a final response rate of 19.8%. Respondents’ home locations were broadly representative of each urban area’s distribution of deprivation scores (S2 Fig).

### Green-space visitation rates and motivations

Participants were asked how often they visit i) the countryside and ii) urban green-spaces on average over the course of a year on an 8 point scale from never to daily (see S1 File for all response options). An open ended question recorded respondents’ main reasons for visiting green-space. These motivations were later categorized as those directly associated with biodiversity knowledge or conservation support (such as watching wildlife, conservation volunteering or gardening) and other motivations (such as fresh air or exercise). Participants were also asked how often they spend time in their garden (nine point scale from no garden, never to daily) and watch or listen to natural history programs (eight point scale from never to daily) on average in a year.

### Biodiversity knowledge

We used photo elicitation of 12 native species and of three habitat types to assess participants’ biodiversity knowledge along three domains: i) species identification (scored 0–12, one point awarded per correct species level identification, and a half mark awarded for correct identification to a group of similar species, e.g. tit rather than blue tit *Cyanistes caeruleus;* see S2 Table for focal species); ii) knowledge of species’ conservation status (scored 0–12, one mark per correct answer; UK Biodiversity Action Plan priority species were coded as being of conservation concern); iii) habitat quality assessment, respondents ranked three images per broad habitat type (arable farmland, wetland and woodland) according to their perceived value for wildlife (scored 0–9, one mark per correct position as assessed by three independent experts, all of which gave identical rankings). Habitat quality assessment was incorporated to include relatively complex mental constructs of biodiversity in addition to more simple scientific facts, as advocated by Fischer & Young [12]. Arable farmland images differed in their field margins’ size and floristic diversity. Wetland images differed in the amount of natural habitat at the land/water interface (i.e. concrete, intensively grazed pasture, natural emergent vegetation). Woodland images differed in tree species diversity and naturalness (conifer plantation, conifer plantation with some silver birch *Betula pendula*, a native broad-leaved woodland). We standardized respondents’ scores for each domain using z scores and conducted a linear principal component analysis (PCA) with direct oblimin rotation using SPSSv21. The three domains loaded on to one dimension which was used as a single unified metric of biodiversity knowledge (eigenvalue 1.91) which accounted for 63.8% of the variation (component loadings: identification 0.86; conservation status 0.78; habitat quality assessment 0.75).

### Support for conservation

We used four domains to measure conservation support. The ‘commitment to environmental sustainability (CESS)’ and ‘willingness to sacrifice (WTS)’ scales relate to environmental attitudes and theoretical behaviors [31, 32]. We used the three question version of CESS which measures pro-environmental attitudes on a five point Likert scale using questions such as “the environment is a low priority for me compared with a lot of other things in my life”. WTS measures theoretical willingness of respondents to change their behavior in order to protect the environment based on five questions on a nine point Likert scale (e.g. “I am willing to give things up that I like doing if they harm the natural environment”). The third domain comprised actual financial conservation support, i.e. monthly expenditure on memberships and donations to environmental and nature conservation organizations. Respondents’ donations were limited to four of the categories provided in the interview (zero, £1–5, £6–10 and £11–20) the mid-points of which were used to calculate expenditure. Finally, as actual donations may be constrained by competing financial pressures, we measured hypothetical financial conservation support; we asked respondents to distribute £600 across six charitable sectors: medical research, human rights, animal welfare, protecting/helping vulnerable people, environmental protection and animal/plant conservation–we summed donations to the last two sectors as a measure of conservation support. 98% of respondents did not donate time to environmental or conservation organizations so these data were not used as they did not allow adequate separation of respondents. Following standardization using z scores a linear PCA, with varimax rotation, identified two axes of conservation support. The WTS and CESS scales loaded onto the first axis (termed behavioral conservation support) with an eigenvalue of 1.88, and explained 47.1% of the variation (S3 Table). The second axis (termed financial conservation support) loaded onto actual and hypothetical financial contributions and explained 22.1% of the variation (S3 Table). We retained this axis to enable more complete exploration of our data although its eigenvalue (0.89) was lower than desirable.

### Socio-economic and demographic information

We recorded participants’ employment status, highest education qualification, tax band (a wealth indicator), age, gender and ethnicity (S4 Table). Categorical principle component analysis (CATPCA) was conducted in SPSSvs21 using employment status, education, tax band, ethnicity and discretized scores (to enable inclusion in CATPCA) of the Index of Multiple Deprivation [30] for the lower super output area containing each participant’s house. The CATPCA identified two axes. Employment, education and tax band loaded on the first axis termed socio-economic status (eigenvalue 1.95, explains 38.9% of the variation; S5 Table). Ethnicity and deprivation scores loaded on the second axis termed ethnicity-deprivation index (eigenvalue 1.49, explains 29.8% of the variation; S5 Table).

### Statistical analysis

A key aspect of our analyses is the relationship between our response variables and visitation rates, which are ordinal data. Such data can be modelled as a continuous or categorical predictor [33]. The former estimates the linear component of a relationship and is recommended by Moses et al. [34]. Treating ordinal data as discrete categories has the advantage of modelling more complicated relationships, but at the cost of using additional degrees of freedom. The diagnostic procedure advocated by Pasta [33] for assessing which approach provided a better fit to the data provided consistent support for treating visit rates as continuous predictors; the results of which are presented in the main text. We, however, also conducted robustness tests where equivalent alternative models that treated visitation rates as ordinal data were constructed (S6 Table). The two sets of analyses yielded qualitatively similar results, indicating that our conclusions are robust to decisions regarding the treatment of ordinal predictors.

To assess the relative use of green-space inside and outside urban areas we classified respondents as visiting i) urban green-space and the countryside equally frequently, ii) urban green-space more frequently or iii) the countryside more frequently and then tested if the distribution of respondents across these three groups differed from random using a chi-squared test.

We used a repeated measures ANOVA (in SPSS vs21) to determine if respondents achieved consistently different biodiversity knowledge scores across the three domains (species identification, knowledge of conservation status and habitat quality). A repeated measures approach was selected as each individual provided multiple biodiversity knowledge scores. Prior to analysis all scores were converted to a percentage, as the maximum potential score varied across domains, and we used a Huynh-Feldt correction, due to inequality of variance, and a Bonferonni post-hoc test.

We used the lme4 package in R [35] to construct linear mixed models to test the hypotheses that i) biodiversity knowledge, behavioral and financial conservation support were positively associated with engaging with nature (measured by green-space visitation rates), and ii) the form of these relationships varied if visits were to urban green-space or the countryside. We used an information theoretic approach and for each response variable constructed three models that contained the following predictors: i) urban green-space visits, social predictors (i.e. socio-economic status, ethnicity-deprivation, age and gender) and city (modelled as a random factor in all cases), ii) countryside visit rates, social predictors and city, and iii) social predictors and city. The correlation between visits to urban green-space and the countryside was too strong (r_s_ = 0.50, *P* < 0.001) to include both predictors in the same model. Social predictors are included in all models as our primary motivation is to take their effects into account rather than testing whether they are associated with our response variables.

To test the hypothesis that conservation support was positively associated with biodiversity knowledge we used the lme4 package to construct mixed models of behavioral and financial conservation support as a function of i) biodiversity knowledge, social predictors and city, and ii) social predictors and city (again as a random factor in both models). We used Akaike Information Criteria scores corrected for small samples sizes (AICc) to compare models, and report the results for all models with ΔAICc values < 4.

Positive associations between green-space visit rates and biodiversity knowledge or conservation support may arise because visiting green-space increases knowledge and support or because people have high visitation rates to green-space as a consequence of activities that are directly associated with biodiversity knowledge and conservation support (e.g. bird-watching, conservation volunteering etc.). Although we are unable to fully disentangle cause and effect, we provide some insight into the direction of these relationships by excluding respondents with such motivations for visiting green-space from analyses; these analyses generated qualitatively similar results (S7 Table) to those conducted using the entire data-set.

It is plausible that other confounding factors drive increased visit rates to green-space whilst also promoting increased biodiversity knowledge or conservation support, thus contributing to a non-causal link between visitation rate and these outcome variables. Three plausible candidates for such confounding factors (although there are others) are garden use, engagement with natural history programs and being a member of a conservation organization. We thus also modeled biodiversity knowledge and conservation support as a function of these explanatory variables (individually) as fixed factors whilst also taking into account social variables and city (as a random factor)–the relationship between financial conservation support and membership of a conservation organization was not modeled as membership fees contribute to the measure of financial support.

Our third and final set of models tested the hypotheses that urbanization (measured as city size and the intensity of local urbanization) reduces visitation rates to green-space, biodiversity knowledge, and conservation support. To describe the overall pattern of association between each response variable and urbanization we modelled response variables as functions of i) local scale urbanization and city (as a random factor in all models), ii) city size and city, and iii) local scale urbanization, city size and city. All these models were constructed using mixed linear regression models in the lme4 package. When modeling visitation rates it was treated as a continuous variable following reference 32. We then repeated these analyses using social variables (socio-economic status, ethnicity-deprivation index, age and gender) as additional predictors to assess if associations between our response variables and urbanization still arise when taking into account variation in residents’ characteristics.

## Results

### Green-space visit rates

**Fig 2:**
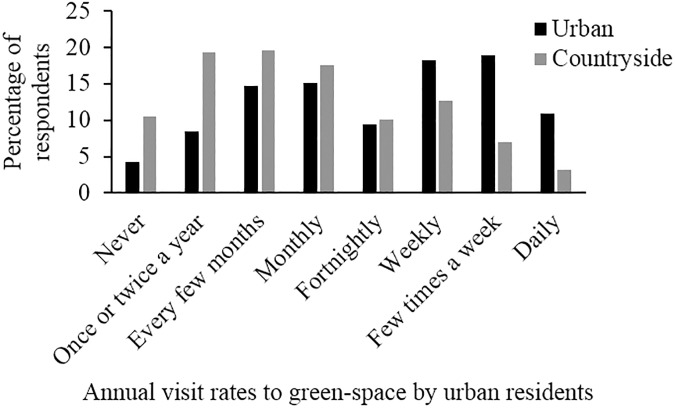
Annual visitation rates of urban residents to urban green-space and the countryside—many urban residents visit green-space, especially the countryside, infrequently.

Most respondents (58%) visited urban green-space more frequently than the countryside, 33% visited urban green-space and the countryside equally frequently, and 10% visited the countryside more frequently; the numbers of people in these three groups differed significantly (χ^2^ = 100.29, *P* < 0.001). 7% of respondents obtained all their exposure to green-space in urban environments, while the equivalent figure for the countryside was 1%. 30% of respondents visited the countryside, and 13% visited urban green-space, no more than twice a year (Fig 2).

### Green-space visit rates and biodiversity knowledge

Respondents were better at assessing habitat quality (mean score 60.96%, SE 1.72), than identifying species (49.65%, SE 1.21) and knowing their conservation status (34.97%, SE 1.32; repeated measures ANOVA F_1.7,491.9_; *P* < 0.0001; all post hoc tests *P* < 0.0001). Biodiversity knowledge (the composite PCA derived metric of these three skills) was positively associated with countryside (but not urban green-space) visit rates and socio-economic status, and negatively associated with ethnicity-deprivation scores; age and gender had little influence (Table 1 and S3A Fig). Garden use, being a member of a conservation organization and time spent watching or listening to natural history programs had negligible influence on biodiversity knowledge with AICc values for these models being at least 7 points higher than that for countryside visits (Table 2).

**Table 1:** Multiple regression models of biodiversity knowledge and conservation support as a function of visitation rates to urban green-space and the countryside.

	*Explanatory variable parameter estimate (95% confidence intervals)*								
*Response variable*	*Countryside visits*	*Urban green-space visits*	*Socio-economic status*	*Ethnicity-deprivation index*	*Age*	*Gender*	*AICc*	*ΔAICc*	*Model weight*
Biodiversity knowledge	0.11 (0.06 to 0.17)		0.27 (0.17 to 0.37)	-0.23 (-0.34 to -0.12)	0.02 (0.01 to 0.02)	0.01 (-0.18 to 0.20)	702.61	0	0.99
Behavioral conservation support	0.12 (0.06 to 0.18)		0.14 (0.02 to 0.25)	0.16 (0.04 to 0.28)	0.00 (-0.01 to 0.00)	-0.19 (-0.41 to 0.02)	763.66	0	0.89
Financial conservation support		0.05 (-0.00 to 0.10)	0.18 (0.06 to 0.11)	-0.10 (-0.21 to 0.01)	0.00 (-0.01 to 0.01)	-0.15 (-0.35 to 0.04)	711.27	0	0.67
"	0.03 (-0.02 to 0.09)		0.20 (0.09 to 0.31)	-0.10 (-0.21 to 0.01)	0.00 (-0.01 to 0.01)	-0.15 (-0.35 to 0.04)	713.49	2.23	0.22
"			0.22 (0.12 to 0.32)	-0.11 (-0.22 to -0.01)	0.00 (-0.01 to 0.01)	-0.13 (-0.33 to 0.06)	715.01	3.74	0.10

Models presented are all those with ΔAICc values < 4; city (random factor) and social variables (fixed factors) were incorporated into all models to control for their influence.

**Table 2:** Regression models of biodiversity knowledge and conservation support as a function of both green-space visitation rates (countryside and urban green-space) and potentially confounding factors (garden use, natural history program engagement and being a member of a conservation organization).

*Response variable*	*Explanatory variable*	*Parameter estimate (95% confidence intervals)*	*AICc*	*ΔAICc*
Biodiversity knowledge	Countryside visits	0.11 (0.06 to 0.17)	702.61	0.00
	Garden use	0.05 (0.01 to 0.09)	709.36	6.75
	Urban greenspace visits	0.06 (0.01 to 0.12)	712.72	10.11
	Conservation organization member	-0.31 (-0.59 to -0.03)	721.62	19.00
	Natural history programs	-0.05 (-0.10 to -0.00)	722.17	19.55
Behavioral conservation support	Countryside visits	0.12 (0.06 to 0.18)	763.66	0.00
	Garden use	0.06 (0.02 to 0.10)	765.81	2.15
	Natural history programs	-0.13 (-0.18 to -0.07)	766.48	2.82
	Urban green-space visits	0.09 (0.03 to 0.15)	767.83	4.17
	Conservation organization member	-0.16 (-0.48 to 0.16)	786.49	22.83
Financial conservation support	Garden use	0.01 (-0.03 to 0.05)	711.26	0.00
	Urban green-space visits	0.05 (-0.00 to 0.10)	711.27	0.01
	Countryside visits	0.03 (-0.02 to 0.09)	713.49	2.24
	Natural history programs	0.02 (-0.03 to 0.07)	716.40	5.14

Social variables (fixed factors) and city (random factor) were incorporated into all models. Membership of conservation organizations contributes to financial conservation support, so the association between the two variables is not assessed.

### Green-space visit rates and conservation support

The monthly total given to all charities through membership subscriptions and donations was £2,914.50. Environmental and conservation charities was the third most popular sector, receiving 8%, with charities supporting vulnerable people (49.8%) and medical research (25.4%) receiving a much greater proportion. Conservation and environmental charities received 17.5% of respondents’ hypothetical spends of £600 per respondent across five charitable sectors, again coming third behind vulnerable people (31.4%) and medical research (29%).

Log transformed financial conservation support was positively associated with visitation rates to urban green-space and the countryside, but the parameter estimates’ 95% confidence intervals overlapped zero (Table 1). We thus conclude that there is negligible evidence for financial conservation support being influenced by green-space visitation rates. Higher socio-economic status was positively associated with financial conservation support while age, gender and ethnicity-deprivation scores had negligible associations (Table 1). The influence of garden use and natural history programs on financial conservation support was negligible as confidence intervals overlapped zero (Table 2).

Behavioral conservation support was positively associated with countryside, but not urban green-space, visit rates and with socio-economic status and ethnicity-deprivation scores; age and gender had negligible influences (Table 1 and S3B Fig). Engaging with natural history programs was negatively associated with behavioral conservation support, and being a member of a conservation group had no influence on behavioral conservation support (Δ AICc = 23, relative to a model containing countryside visit rates, Table 2). Behavioral conservation support was positively associated with garden use but the evidence for this relationship was lower than that for an association with countryside visit rates (Δ AICc > 2) and the parameter estimate was substantially smaller despite both predictors having equivalent scales (Table 2).

### Association between biodiversity knowledge and conservation support

Behavioral and financial conservation support were positively associated with biodiversity knowledge, with the magnitude of the parameter estimate in the behavioral support model being double that of the financial support model (Table 3). Behavioral conservation support was positively associated with the ethnicity-deprivation index; whilst also positively associated with socio-economic status, 95% confidence intervals overlapped zero suggesting negligible influence (Table 3). Financial conservation support was positively associated with socio-economic status and negatively associated with the ethnicity-deprivation index. Age and gender had negligible influence on either measure of conservation support.

**Table 3:** Multiple regression models of conservation support as a function of biodiversity knowledge.

	*Explanatory variable parameter estimate (95% confidence intervals)*							
*Response variable*	*Biodiversity knowledge*	*Socio-economic status*	*Ethnicity-deprivation index*	*Age*	*Gender*	*AICc*	*ΔAICc*	*Model weight*
Behavioral conservation support	0.36 (0.24 to 0.49)	0.09 (-0.03 to 0.20)	0.21 (0.09 to 0.33)	-0.01 (-0.02 to 0.00)	-0.18 (-0.39 to 0.03)	756.22	0	0.99
Financial conservation support	0.18 (0.06 to 0.29)	0.16 (0.05 to 0.27)	-0.07 (-0.07 to -0.06)	0.00 (-0.01 to 0.00)	-0.13 (-0.33 to 0.06)	711.27	0	0.67

Models presented are all those with ΔAICc values < 4; city (random factor) and social variables (fixed factors) were incorporated into all models to control for their influence.

### Urbanization and green-space visit rates

More intense urbanization around respondents’ homes was negatively associated with countryside visitation rates when accounting for social factors (S8 Table and S5A Fig) and when excluding them from models (Table 4 and S4A Fig). There was no association between city size and countryside visits both when including (S8 Table) and excluding social factors (Table 4). Countryside visitation rates were positively associated with socio-economic status and negatively related to the ethnicity-deprivation index; age and gender had negligible influence (S8 Table).

**Table 4:** Multiple regression models of green-space visitation rates, biodiversity knowledge, and conservation support as a function of local scale urbanization intensity and city size, without controlling for social factors (see S8 Table for equivalent models that take social factors into account).

	*Explanatory variable parameter estimate (95% confidence intervals)*				
*Response variable*	*Local urbanization*	*City size (small)*	*AICc*	*ΔAICc*	*Model weight*
Countryside visit rate	-0.22 (-0.33 to -0.12)		1161.49	0.00	0.54
"	-0.21 (-0.32 to -0.11)	0.34 (-0.14 to 0.82)	1161.82	0.32	0.46
Urban green-space visit rate	-0.96 (-0.21 to 0.02)		1208.18	0.00	0.41
" ^*b*^			1208.88	0.70	0.29
Biodiversity knowledge	-0.09 (-0.14 to -0.03)		788.99	0.00	0.62
"	-0.08 (-0.14 to -0.03)	0.24 (-0.24 to 0.73)	790.14	1.15	0.35
Behavioral conservation support		-0.33 (-0.56 to -0.10)	797.20	0.00	0.61
"	0.02 (-0.04 to 0.08)	-0.30 (-0.54 to -0.06)	798.67	1.48	0.29
Financial conservation support	-0.07 (-0.12 to -0.02)		725.28	0.00	0.68
"	-0.07 (-0.12 to -0.02)	0.00 (-0.30 to 0.31)	727.35	2.07	0.11

We present all models with ΔAICc values < 4 of the best performing model except when these models have higher ΔAICc values than a model that only contains city as a random effect.

^b^This model only contains city as a random effect.

When social factors were excluded from analyses, urban green-space visit rates were not associated with city size or local urbanization (whilst retained in the top model the AICc value was very close to the null model and 95% confidence intervals overlapped zero; Table 4). When accounting for social factors, however, participants from larger cities reported higher visitations rates to urban green-spaces than those from smaller urban areas (S8 Table and S5B Fig) though the association with local urbanization was still negligible (S8 Table). Visitation rates to urban green-space were positively associated with socio-economic status and negatively related to the ethnicity-deprivation index; age and gender had negligible influence (S8 Table).

### Urbanization and biodiversity knowledge

Biodiversity knowledge was negatively associated with urbanization at the local scale when excluding social factors from analyses (Table 4 and S4B Fig) but not when including them (S8 Table); city size consistently had no influence (95% parameter estimates overlapped zero; Table 4 and S8 Table). Biodiversity knowledge was positively associated with socio-economic status and age, and negatively associated with the ethnicity-deprivation index while gender had no influence (S8 Table).

### Urbanization and conservation support

Behavioral support for conservation was greatest amongst respondents from larger urban areas when including (S8 Table and S5C Fig) and excluding (Table 4 and S4C Fig) social factors from models. In both cases the intensity of local urbanization was negligible (parameter estimate’s 95% confidence intervals overlapped zero). Behavioral conservation support was positively associated with socio-economic status, with other social factors having negligible influence (S8 Table).

Financial conservation support was negatively associated with urbanization at the local scale when excluding socio-demographic variables from models (Table 4) though the predictive power of the model was low (S4C Fig). When social factors were incorporated, the association between financial conservation support and local urbanization became negligible (S8 Table). City size consistently had negligible influence on financial conservation support (Table 4 and S8 Table). Socio-economic status was positively associated with financial support while all other social factors had negligible influence (S8 Table).

## Discussion

We find that many urban residents in the UK visit green-space infrequently, which is compatible with previous studies [23, 36]. This low engagement was particularly notable when considering visits to the countryside, with one third of urban residents visiting twice a year or less, and the majority of urban residents visiting urban green-space more frequently than the countryside. Respondents’ knowledge of species identification and conservation status was also low (nearly one in two participants were unable to name more than half of the species and 65% knew the conservation status of less than half the species). These findings concur with other evidence that the general public’s biodiversity knowledge is poor [10]. However, respondents were significantly more capable of assessing the relative biodiversity value of different habitat types than naming species or assessing their conservation status. This is encouraging as recognition of habitat quality is often a prerequisite for conservation action, e.g. habitat management. Assessing habitat quality is a complex mental process that requires understanding variation in species’ habitat requirements. Our results thus offer some support to claims that studies reporting poor biodiversity knowledge are influenced by focusing on precise scientific knowledge rather than more general constructs and understanding of ecological processes [12, 13] although further work is required to determine exactly how participants judge habitat quality to confirm this. Interestingly, however, all three measures of biodiversity knowledge loaded onto the same axis during principal component analysis, thus studies reporting just one of these metrics are still likely to provide a useful surrogate for a wider holistic metric of biodiversity knowledge.

The CES and WTS scales assess participants’ self-reported engagement in pro-environmental behaviors whilst taking other constraints and priorities into account [31, 32]. These scales are thus designed to address the ‘action gap’ between pro-environmental attitudes and pro-environmental behaviors [17, 37]. Despite this participants’ CES and WTS scores, whilst being positively associated with each other, did not load on to the same PCA axis as actual and hypothetical donations to conservation. This may partly arise from variation in respondents’ financial situations but may also occur due to contextual variation in the way participants interpret specific questions, as documented for other related scales (e.g. the New Ecological Paradigm; [38]). Another key factor that probably contributes to the limited association between measures of participants’ behavioral support for conservation (CES and WTS scores) and financial conservation support is that people prioritize their own finances and donations to other charitable sectors, rather than conservation. This is evidenced by respondents’ substantially greater donations to medical research charities and those that help vulnerable people compared to conservation. Despite these limitations the CES and WTS scales do capture useful information on respondents’ pro-environmental attitudes and self-reported behaviors, and perform well compared to alternative scales [31, 32].

Our research builds upon a small number of important earlier studies finding that visits to green-space are positively associated with species identification skills [39, 40], and studies reporting a mix of positive and negligible associations between visiting green-spaces and conservation support (e.g. [19, 41, 42]). In particular we incorporated a more holistic measure of biodiversity knowledge, took visitor motivations into account, selected respondents outside green-spaces in an unbiased manner and contrasted the effects of visiting urban green-space and the countryside. We found evidence for our core hypothesis that people who visit green-spaces more regularly have greater levels of biodiversity knowledge and behavioral conservation support. We find that these associations remain when taking into account potentially confounding variables relating to motivations for visiting green-space that are associated with nature. We also find that biodiversity knowledge and conservation support are much more strongly associated with green-space visitation rates than other potentially confounding variables, i.e. garden use and engagement with natural history programs. Observational studies cannot, however, unambiguously demonstrate causality. It thus remains plausible that the positive relationships we describe arise in part because people with greater biodiversity knowledge and conservation support visit green-space more regularly, rather than visiting green-space increasing knowledge and conservation support. Indeed, we consider it likely that the associations we describe arise through both of these mechanisms due to a positive feedback loop, at least in some respondents—with increased visits to green-space promoting an interest in and knowledge of biodiversity and support for conservation, which in turn further increase the desire to visit green-space and experience nature.

Crucially we found that higher levels of biodiversity knowledge and behavioral conservation support were associated with more frequent visits to the countryside, and not urban green-space. Our findings thus provide support for the hypothesis of a concerning ecosystem service provision gap with urban green-space providing negligible benefits relative to the countryside (Fig 1). We suspect that this provisioning gap arises because visiting urban green-spaces exposes respondents to the more limited range of species that occur in such environments relative to the countryside [24]. Indeed, other studies find that the magnitude of cultural ecosystem services provided by urban green-space is related to perceived or actual species richness [42], which suggests that the ecosystem service provisioning gap that we identify between urban green-space and the countryside may apply to other cultural services.

Across our range of city sizes, which are representative of many found throughout Europe and include some of the largest urban areas in England, we found no evidence that residents from larger cities visit the countryside less or have reduced biodiversity knowledge or support for conservation than those from smaller cities. In fact, behavioral support for conservation was higher amongst people from larger cities. This pattern cannot be driven by higher educational standards in urban areas, as previously suggested by Huddart-Kennedy et al. [22], as our analysis took educational status and other social factors into account. It is plausible that environmental degradation generated by large scale urbanization promotes greater support for conservation [20], but this also seems somewhat unlikely to drive our findings as we did not find that people living in more intensely urbanized areas had higher levels of conservation support. Those respondents living in intensely urbanized environments visited the countryside less often, even when taking social factors into account. Whilst some studies report lower biodiversity knowledge in urban residents than those living in the countryside, no studies appear to have assessed associations between urbanization intensity and biodiversity knowledge amongst urban residents [16, 40]. It is thus notable that we found residents in highly urbanized locations had lower biodiversity knowledge. This relationship was partly driven by social factors but may also be linked to their lower countryside visitation rates and the reduced biodiversity that occurs in highly urbanized areas [43].

We provide a vital first step in the provision of evidence that engaging with nature by visiting green-spaces is associated with enhanced biodiversity knowledge and conservation support as predicted by conservation policies that aim to increase peoples’ exposure to green-space. These relationships, however, only arose from visiting the countryside and not urban green-spaces. We have thus uncovered a previously undescribed mechanism through which urban development may reduce biodiversity knowledge and conservation support. This is concerning as many urban dwellers visit the countryside very infrequently. Larger cities were not associated with reduced biodiversity knowledge and conservation support, but the intensity of urban development around respondent’s homes was associated with reduced countryside visit rates and biodiversity knowledge. Conservation policies that aim to maximize urban residents’ abilities to develop and maintain biodiversity knowledge and support for conservation should thus focus on creating less dense cities, rather than smaller but more intensely urbanized ones, whilst enhancing access to the countryside.

## Supporting information

S1 Fig Urbanization scoring by satellite imagery. Satellite imagery depicting the extremes of local urbanization scores included within the study with a) the postcode with the lowest surrounding local urbanization score (-6.21) and b) the postcode with the highest surrounding local urbanization score (3.05). Scores are calculated using image recognition software (Seress et al. 2014) to generate a single metric of urbanization based on a semi-automated assessment of the area of buildings, roads, other impervious surface, and vegetated green-space from google earth aerial photographs. Ground truthing confirmed that images accurately represented land cover at the time of the door-to-door surveys.
(TIF)

S2 Fig Index of Multiple Deprivation scores. The distributions of the Index of Multiple Deprivation scores for (a) all Lower Super Output Areas (LSOAs) within 3 km of each urban area center and (b) the LSOAs within which the survey respondents live for the same urban area. Distributions of both are similar indicating that questionnaire participants were selected in an unbiased manner.
(TIF)

S3 Fig Green-space visit relationships with biodiversity knowledge and conservation support when controlling for social factors. Relationships between countryside visitation rates and (a) biodiversity knowledge (a PCA derived score combining knowledge of species’ identification, conservation status and habitat quality assessment), and (b) behavioral conservation support (a PCA derived score combining commitment to the environment and willingness to sacrifice scales). Grey bars represent raw data; black dots are predicted scores from linear mixed-effects models that include social variables as fixed factors, and city as a random factor. Error bars represent standard errors. See Table 1 for full results.
(TIF)

S4 Fig Urbanization relationships with green-space visits, biodiversity knowledge and conservation support without controlling for social factors. Associations between (a) countryside visitation rate and local urbanization score (urbanization intensity near respondents’ homes, higher scores represent greater urbanization), (b) biodiversity knowledge and local urbanization score, (c) behavioral conservation support and size of urban area, and (d) financial conservation support and local urbanization score. Grey bars represent raw data; black dots are predicted scores from linear mixed-effects models that include city as a random factor but not social variables. Error bars represent standard errors. Note the poor fit of the predicted and observed financial conservation support data. See Table 4 for full results.
(TIF)

S5 Fig Urbanization relationships with green-space visits, biodiversity knowledge and conservation support when controlling for social factors. Associations between (a) countryside visitation rates and local urbanization score, (b) urban green-space visitation rate and size of the urban area, and (c) behavioral conservation support and size of the urban area. Grey bars represent raw data; black dots are predicted scores from linear mixed-effects models that include social variables as fixed factors, and city as a random factor. See S8 Table for full results.
(TIF)

S1 Table Number of completed questionnaires per city. (DOCX)

S2 Table Biodiversity knowledge indicator species list. The twelve native species used to assess respondents’ knowledge of species identification and conservation status (Biodiversity Action Plan priority species). All species are distributed across the entire survey area, enabling direct comparison between different locations, but within each taxonomic group two species are common in urban areas and two are typically confined to rural areas. Two bird and two mammal species were BAP priority species. No BAP plants were included as all such species have very local distributions rendering it impossible for respondents from all survey locations to have had equal opportunity of encountering them.
(DOCX)

S3 Table Conservation support PCA. Conservation support was measured using four domains which loaded onto two axes in a linear PCA (varimax rotation). Pro-environmental attitudes and reported behavior loaded onto the first axis (eigenvalue 1.88) and is termed behavioral conservation support. Financial donations loaded primarily onto the second axis and (despite its lower than ideal eigenvalue of 0.89) is retained to enable more complete exploration of conservation support and due to its very strong loading onto actual financial contributions.
(DOCX)

S4 Table Socio-demographic indicator answer scales. Participants were also asked for their postcodes.
(DOCX)

S5 Table Socio-demographic PCA. A categorical PCA of five socio-demographic indicators identified two axes. Education, employment and tax band loaded primarily onto the first axis (eigenvalue 1.95) and forms our indicator of socio-economic status. Ethnicity and the Index of Multiple Deprivation (which is a continuous variable and was thus discretized prior to analysis) loaded primarily onto the second axis (eigenvalue 1.49).
(DOCX)

S6 Table Regression model output for green-space visitation rate impacts on biodiversity knowledge and conservation support when visit rates are modelled as categorical. The models confirm the results obtained when modelling visitation rates as continuous variables (Table 1), i.e. that biodiversity knowledge and behavioral conservation support are positively associated with countryside visitation rates but have negligible associations with visits to urban green-space, and that financial conservation support is not strongly associated with visits to the countryside or urban green-space. Models presented are all those with ΔAICc values < 4; city (random factor) and social variables (fixed factors) were incorporated into all models to control for their influence.
(DOCX)

S7 Table Regression model output for green-space visitation rate impacts on biodiversity knowledge and conservation support when excluding participants motivated by nature related activities. Models presented are all those with ΔAICc values < 4; city (random factor) and social variables (fixed factors) were incorporated into all models to control for their influence.
(DOCX)

S8 Table Regression model output for urbanization impacts on visit rates to green-spaces, biodiversity knowledge and conservation support when controlling for social factors. Models presented are all those with ΔAICc values < 4 of the best performing model except when these models have higher ΔAICc values than a model that only contains city; city (random factor) and social variables (fixed factors) were incorporated into all models to control for their influence. City size is a binary fixed factor with parameter estimates for large cities set to zero.
(DOCX)

S1 File Questionnaire. (PDF)
