# Supplementary figures and images for: Contrasting effects of visiting urban green-space and the countryside on biodiversity knowledge and conservation support
Source: PLoS One. 2017 Mar 23;12(3):e0174376. doi: 10.1371/journal.pone.0174376 (PMC5363982; doi:10.1371/journal.pone.0174376)

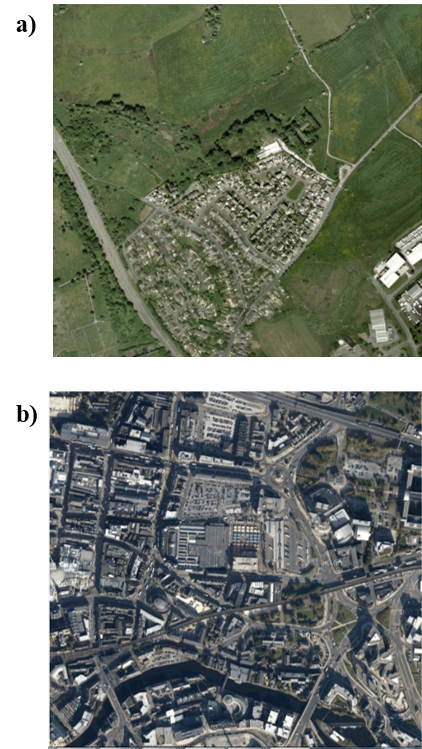

Supplement: S1 Fig — Satellite imagery depicting the extremes of local urbanization scores included within the study with a) the postcode with the lowest surrounding local urbanization score (-6.21) and b) the postcode with the highest surrounding local urbanization score (3.05). Scores are calculated using image recognition software (Seress et al. 2014) to generate a single metric of urbanization based on a semi-automated assessment of the area of buildings, roads, other impervious surface, and vegetated green-space from google earth aerial photographs. Ground truthing confirmed that images accurately represented land cover at the time of the door-to-door surveys. (TIF) [file pone.0174376.s001.tif]

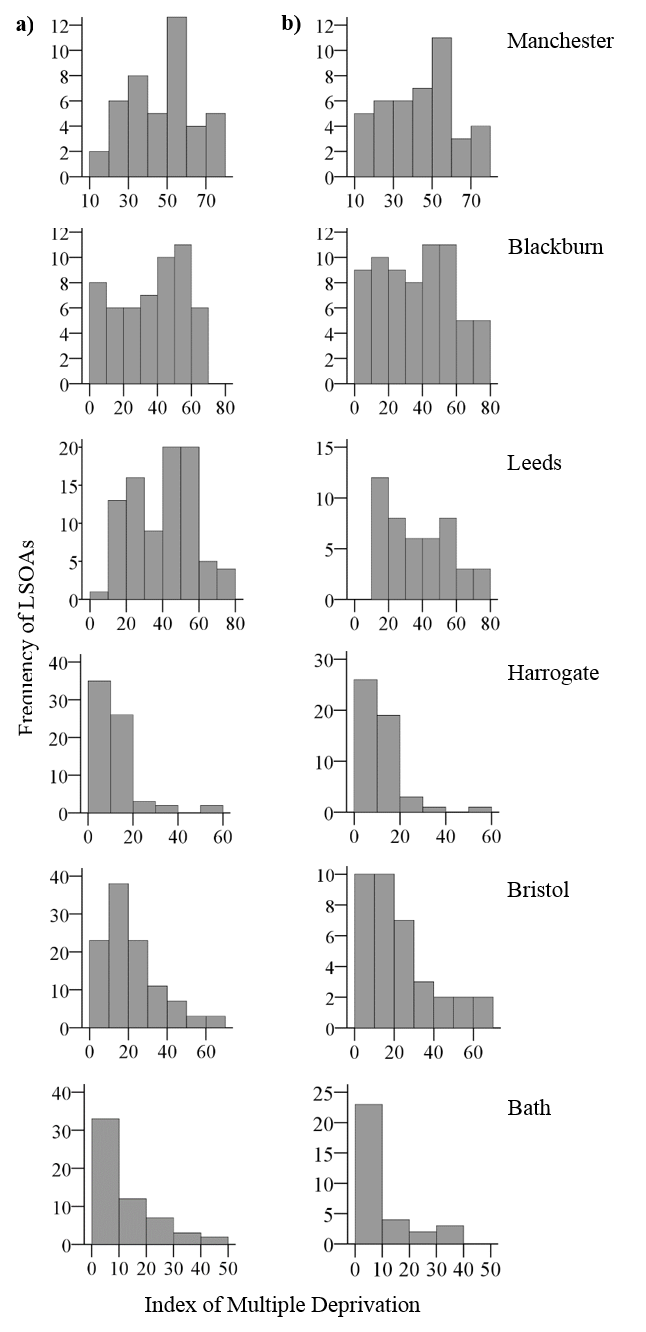

Supplement: S2 Fig — The distributions of the Index of Multiple Deprivation scores for (a) all Lower Super Output Areas (LSOAs) within 3 km of each urban area center and (b) the LSOAs within which the survey respondents live for the same urban area. Distributions of both are similar indicating that questionnaire participants were selected in an unbiased manner. (TIF) [file pone.0174376.s002.tif]

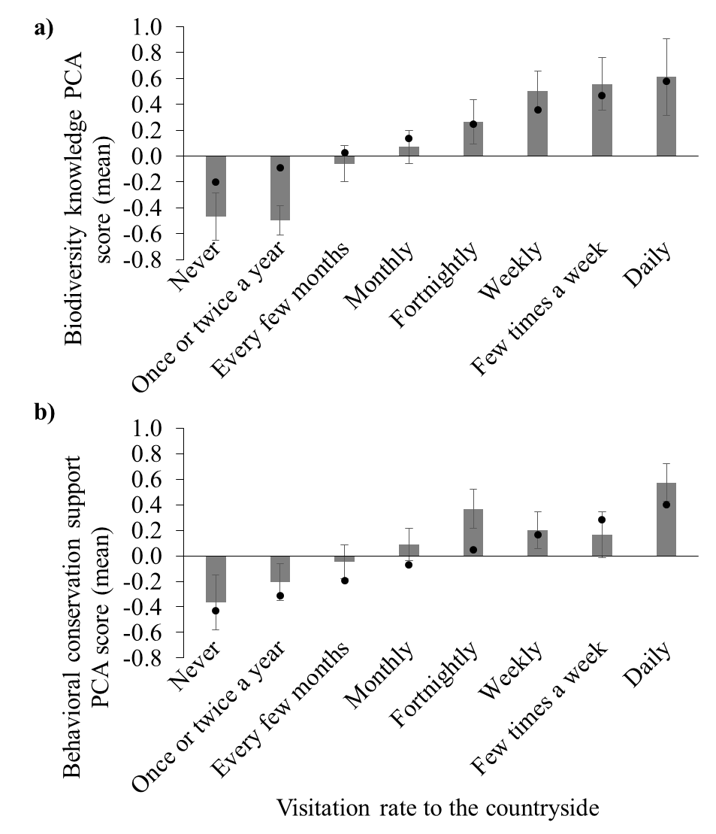

Supplement: S3 Fig — Relationships between countryside visitation rates and (a) biodiversity knowledge (a PCA derived score combining knowledge of species’ identification, conservation status and habitat quality assessment), and (b) behavioral conservation support (a PCA derived score combining commitment to the environment and willingness to sacrifice scales). Grey bars represent raw data; black dots are predicted scores from linear mixed-effects models that include social variables as fixed factors, and city as a random factor. Error bars represent standard errors. See Table 1 for full results. (TIF) [file pone.0174376.s003.tif]

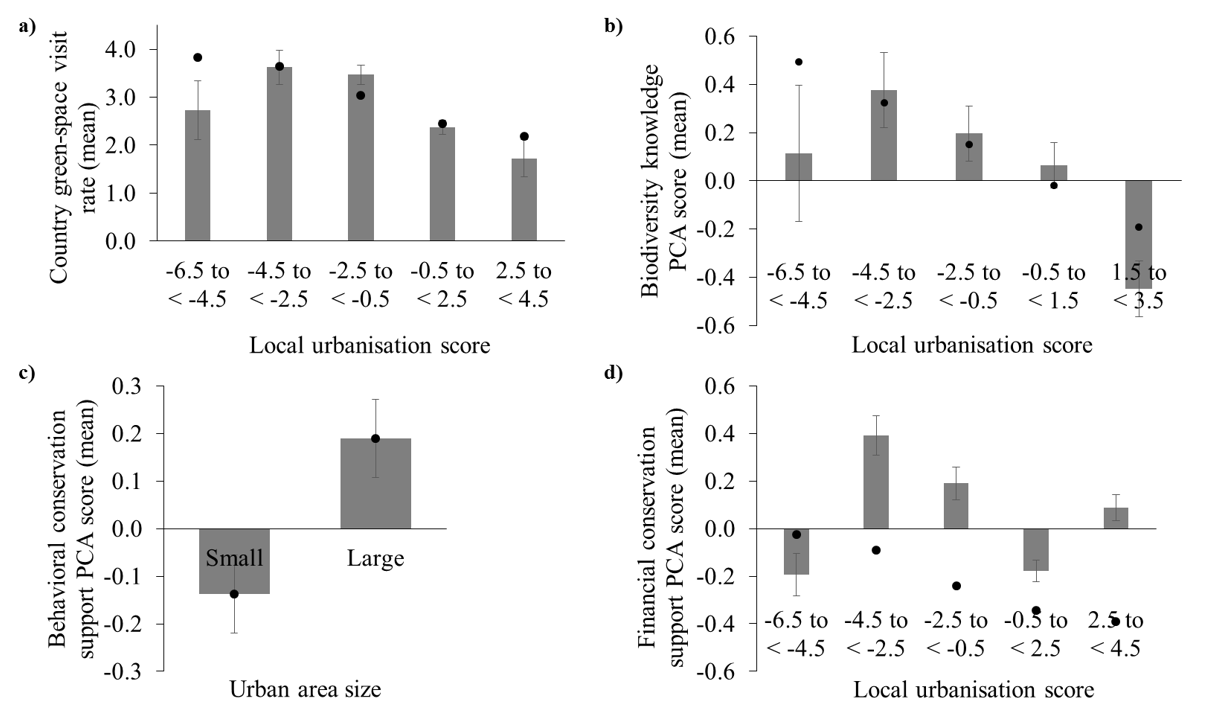

Supplement: S4 Fig — Associations between (a) countryside visitation rate and local urbanization score (urbanization intensity near respondents’ homes, higher scores represent greater urbanization), (b) biodiversity knowledge and local urbanization score, (c) behavioral conservation support and size of urban area, and (d) financial conservation support and local urbanization score. Grey bars represent raw data; black dots are predicted scores from linear mixed-effects models that include city as a random factor but not social variables. Error bars represent standard errors. Note the poor fit of the predicted and observed financial conservation support data. See Table 4 for full results. (TIF) [file pone.0174376.s004.tif]

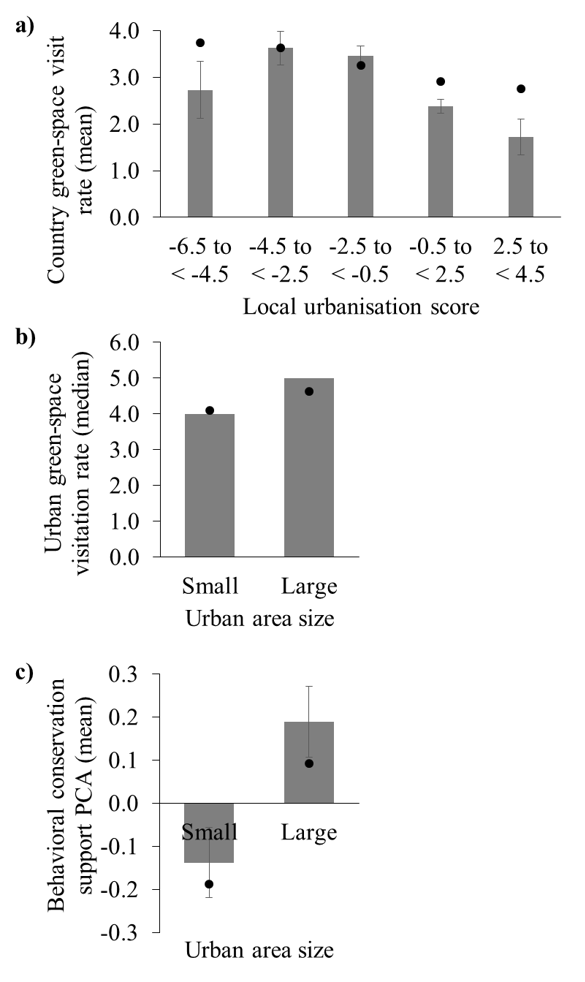

Supplement: S5 Fig — Associations between (a) countryside visitation rates and local urbanization score, (b) urban green-space visitation rate and size of the urban area, and (c) behavioral conservation support and size of the urban area. Grey bars represent raw data; black dots are predicted scores from linear mixed-effects models that include social variables as fixed factors, and city as a random factor. See S8 Table for full results. (TIF) [file pone.0174376.s005.tif]
