# Supplementary material for: Contrasting effects of visiting urban green-space and the countryside on biodiversity knowledge and conservation support
Source: PLoS One. 2017 Mar 23;12(3):e0174376. doi: 10.1371/journal.pone.0174376 (PMC5363982; doi:10.1371/journal.pone.0174376)
Supplement: S1 Table — (DOCX) [file pone.0174376.s006.docx]

| *Large urban area* | | *Small urban area* | |
| --- | --- | --- | --- |
| *Name* | *No. Questionnaires* | *Name* | *No. Questionnaires* |
| Manchester | 41 | Blackburn | 55 |
| Leeds | 53 | Harrogate | 68 |
| Bristol | 37 | Bath | 32 |
