# Supplementary material for: Contrasting effects of visiting urban green-space and the countryside on biodiversity knowledge and conservation support
Source: PLoS One. 2017 Mar 23;12(3):e0174376. doi: 10.1371/journal.pone.0174376 (PMC5363982; doi:10.1371/journal.pone.0174376)
Supplement: S2 Table — The twelve native species used to assess respondents’ knowledge of species identification and conservation status (Biodiversity Action Plan priority species). All species are distributed across the entire survey area, enabling direct comparison between different locations, but within each taxonomic group two species are common in urban areas and two are typically confined to rural areas. Two bird and two mammal species were BAP priority species. No BAP plants were included as all such species have very local distributions rendering it impossible for respondents from all survey locations to have had equal opportunity of encountering them. (DOCX) [file pone.0174376.s007.docx]

| *English name* | *Scientific name* | *Common in urban areas?* | *BAP species* |
| --- | --- | --- | --- |
| Blue tit | *Cyanistes caeruleus* | Yes | No |
| Starling | *Sturnus vulgaris* | Yes | Yes |
| Nuthatch | *Sitta europaea* | No | No |
| Linnet | *Carduelis cannabina* | No | Yes |
| Red Fox | *Vulpes vulpes* | Yes | No |
| Hedgehog | *Erinaceus europaeus* | Yes | Yes |
| Mole | *Talpa europaea* | No | No |
| Water vole | *Arvicola amphibius* | No | Yes |
| Daisy | *Bellis perennis* | Yes | No |
| Dandelion | *Taraxacum officinale* | Yes | No |
| Wood sorrel | *Oxalis acetosella* | No | No |
| Yellow Rattle | *Rhinanthus minor* | No | No |
