# Supplementary material for: Contrasting effects of visiting urban green-space and the countryside on biodiversity knowledge and conservation support
Source: PLoS One. 2017 Mar 23;12(3):e0174376. doi: 10.1371/journal.pone.0174376 (PMC5363982; doi:10.1371/journal.pone.0174376)
Supplement: S3 Table — Conservation support was measured using four domains which loaded onto two axes in a linear PCA (varimax rotation). Pro-environmental attitudes and reported behavior loaded onto the first axis (eigenvalue 1.88) and is termed behavioral conservation support. Financial donations loaded primarily onto the second axis and (despite its lower than ideal eigenvalue of 0.89) is retained to enable more complete exploration of conservation support and due to its very strong loading onto actual financial contributions. (DOCX) [file pone.0174376.s008.docx]

| *Conservation support indicator* | *Component loadings* | |
| --- | --- | --- |
|  | *Axis 1* | *Axis 2* |
| Commitment to environmental sustainability | 0.778 | 0.241 |
| Willingness to sacrifice | 0.868 | 0.012 |
| Actual financial contribution to conservation (memberships and donations) | 0.027 | 0.927 |
| Hypothetical financial contribution to conservation (£600 distributed across at least one of 6 charitable sectors) | 0.452 | 0.538 |
