# Supplementary material for: Contrasting effects of visiting urban green-space and the countryside on biodiversity knowledge and conservation support
Source: PLoS One. 2017 Mar 23;12(3):e0174376. doi: 10.1371/journal.pone.0174376 (PMC5363982; doi:10.1371/journal.pone.0174376)
Supplement: S4 Table — Participants were also asked for their postcodes. (DOCX) [file pone.0174376.s009.docx]

| *Socio-economic / demographic indicator* | *Answer categories* |
| --- | --- |
| Employment | Retired, Unemployed, Full-time education, Full-time paid employment, Part-time paid employment, Self-employed, Home maker/bringing up family |
| Education | None, O level/GCSE or equivalent, A level or equivalent, Undergraduate degree, Higher degree, Vocational qualification (level if known) |
| Tax band | No tax (<£9,440 taxable income), Basic rate (£9,440-£32,010), Higher rate (£32,011-£150,000), Top rate (£>150,000) |
| Ethnicity | Office for National Statistics guidelines were used for ethnic group selection (http://ons.gov.uk/ons/guide-method/measuring-equality/equality/ethnic-nat-identity-religion/ethnic-group/index.html) but for analysis purposes these were categorized as white British, white non-British and non-white |
| Age | 16-18, 19-24, 25-34, 35-44, 45-54, 55-64, 65-74, 75-84, 85-94, 94+ |
| Gender | Male, Female |
