# Supplementary material for: Contrasting effects of visiting urban green-space and the countryside on biodiversity knowledge and conservation support
Source: PLoS One. 2017 Mar 23;12(3):e0174376. doi: 10.1371/journal.pone.0174376 (PMC5363982; doi:10.1371/journal.pone.0174376)
Supplement: S5 Table — A categorical PCA of five socio-demographic indicators identified two axes. Education, employment and tax band loaded primarily onto the first axis (eigenvalue 1.95) and forms our indicator of socio-economic status. Ethnicity and the Index of Multiple Deprivation (which is a continuous variable and was thus discretized prior to analysis) loaded primarily onto the second axis (eigenvalue 1.49). (DOCX) [file pone.0174376.s010.docx]

| *Social indicators* | *Component loadings* | |
| --- | --- | --- |
|  | *Axis 1* | *Axis 2* |
| Highest level qualification | 0.819 | 0.102 |
| Employment status | 0.720 | 0.448 |
| Tax band | 0.719 | 0.155 |
| Ethnicity | -0.231 | 0.834 |
| Index of Multiple Deprivation | -0.434 | 0.749 |
