# Supplementary material for: Contrasting effects of visiting urban green-space and the countryside on biodiversity knowledge and conservation support
Source: PLoS One. 2017 Mar 23;12(3):e0174376. doi: 10.1371/journal.pone.0174376 (PMC5363982; doi:10.1371/journal.pone.0174376)
Supplement: S6 Table — The models confirm the results obtained when modelling visitation rates as continuous variables (Table 1), i.e. that biodiversity knowledge and behavioral conservation support are positively associated with countryside visitation rates but have negligible associations with visits to urban green-space, and that financial conservation support is not strongly associated with visits to the countryside or urban green-space. Models presented are all those with ΔAICc values < 4; city (random factor) and social variables (fixed factors) were incorporated into all models to control for their influence. (DOCX) [file pone.0174376.s011.docx]

|  | *Explanatory variable parameter estimate (95% confidence interval)* | | | | | | | | | | | |  |  |  |
| --- | --- | --- | --- | --- | --- | --- | --- | --- | --- | --- | --- | --- | --- | --- | --- |
| *Response variable* | *Green-space visit type* | *Green-space visit rate* | | | | | | | *Socio-economic status* | *Ethnicity-deprivation index* | *Age* | *Gender* | *AICc* | *ΔAICc* | *Model weight* |
|  |  | *Once/twice a year* | *Every few months* | *Monthly* | *Fortnightly* | *Several times a week* | *Weekly* | *Daily* |  |  |  |  |  |  |  |
| Biodiversity knowledge | Countryside | 0.07 (-0.29 to 0.43) | 0.26 (-0.11 to 0.63) | 0.26 (-0.13 to 0.65) | 0.46 (0.03 to 0.89) | 0.57 (0.16 to 0.98) | 0.69 (0.23 to 1.16) | 0.66 (0.04 to 1.27) | 0.27 (0.16 to 0.38) | -0.23 (-0.34 to -0.12) | 0.02 (0.01 to 0.02) | 0.01 (-0.18 to 0.20) | 714.82 | 0 | 0.98 |
| Behavioural conservation support | Countryside | 0.02 (-0.39 to 0.43) | 0.18 (-0.24 to 0.59) | 0.30 (-0.14 to 0.74) | 0.64 (0.15 to 1.13) | 0.53 (0.06 to 1.00) | 0.51 (-0.02 to 1.04) | 0.92 (0.23 to 1.61) | 0.13 (0.01 to 0.25) | 0.17 (0.04 to 0.29) | 0.00 (-0.01 to 0.00) | -0.22 (-0.43 to 0.00) | 774.19 | 0 | 0.70 |
|  | Urban green-space | 0.09 (-0.56 to 0.73) | 0.10 (-0.49 to 0.69) | 0.19 (-0.41 to 0.79) | 0.64 (0.00 to 1.27) | 0.33 (-0.26 to 0.93) | 0.64 (0.04 to 1.24) | 0.50 (-0.12 to 1.13) | 0.14 (0.02 to 0.26) | 0.14 (0.02 to 0.26) | 0.00 (-0.01 to 0.00) | -0.15 (-0.37 to 0.07) | 775.92 | 1.73 | 0.30 |
| Financial conservation support |  |  |  |  |  |  |  |  | 0.22 (0.12 to 0.32) | -0.11 (-0.22 to -0.01) | 0.00 (-0.01 to 0.01) | -0.13 (-0.33 to 0.06) | 715.01 | 0 | 0.84 |
