# Supplementary material for: Contrasting effects of visiting urban green-space and the countryside on biodiversity knowledge and conservation support
Source: PLoS One. 2017 Mar 23;12(3):e0174376. doi: 10.1371/journal.pone.0174376 (PMC5363982; doi:10.1371/journal.pone.0174376)
Supplement: S7 Table — Models presented are all those with ΔAICc values < 4; city (random factor) and social variables (fixed factors) were incorporated into all models to control for their influence. (DOCX) [file pone.0174376.s012.docx]

|  | *Explanatory variable parameter estimate (95% confidence intervals)* | | | | | | *AICc* | *ΔAICc* | *Model weight* |
| --- | --- | --- | --- | --- | --- | --- | --- | --- | --- |
| *Response variable* | *Countryside visit rate* | *Urban green-space visit rate* | *Socio-economic status* | *Ethnicity-deprivation index* | *Age* | *Gender* |  |  |  |
| Biodiversity knowledge | 0.10  (0.04 to 0.15) |  | 0.30  (0.19 to 0.41) | -0.22  (-0.33 to -0.10) | 0.02  (-0.04 to 0.08) | 0.05  (-0.15 to 0.25) | 650.15 | 0 | 0.94 |
| Behavioral conservation support | 0.10  (0.03 to 0.16) |  | 0.14  (0.02 to 0.27) | 0.13  (0.01 to 0.26) | 0.00  (-0.01 to 0.00) | -0.22  (-0.44 to 0.01) | 697.81 | 0 | 0.61 |
| " |  | 0.09  (0.03 to 0.15) | 0.14  (0.01 to 0.26) | 0.12  (0.00 to 0.24) | 0.00  (-0.01 to 0.00) | -0.20  (-0.42 to 0.02) | 698.74 | 0.93 | 0.39 |
| Financial conservation support |  | 0.05  (0.00 to 0.11) | 0.17  (0.06 to 0.28) | -0.10  (-0.22 to 0.01) | 0.00  (-0.01 to 0.00) | -0.11  (-0.31 to 0.09) | 637.16 | 0 | 0.58 |
| " | 0.05  (-0.01 to 0.10) |  | 0.18  (0.06 to 0.28) | -0.10  (-0.22 to 0.01) | 0.00  (-0.01 to 0.00) | -0.09  (-0.31 to 0.08) | 638.28 | 1.10 | 0.33 |
| " |  |  | 0.21  (0.10 to 0.31) | -0.12  (-0.24 to -0.01) | 0.00  (-0.01 to 0.01) | -0.09  (-0.29 to 0.11) | 640.96 | 3.80 | 0.09 |
