# Supplementary material for: Contrasting effects of visiting urban green-space and the countryside on biodiversity knowledge and conservation support
Source: PLoS One. 2017 Mar 23;12(3):e0174376. doi: 10.1371/journal.pone.0174376 (PMC5363982; doi:10.1371/journal.pone.0174376)
Supplement: S8 Table — Models presented are all those with ΔAICc values < 4 of the best performing model except when these models have higher ΔAICc values than a model that only contains city; city (random factor) and social variables (fixed factors) were incorporated into all models to control for their influence. City size is a binary fixed factor with parameter estimates for large cities set to zero. (DOCX) [file pone.0174376.s013.docx]

|  | *Explanatory variable parameter estimate (95% confidence intervals)* | | | | | | *AICc* | *ΔAICc* | *Model weight* |
| --- | --- | --- | --- | --- | --- | --- | --- | --- | --- |
| *Response variable* | *Local urbanisation* | *City size (small)* | *Socio-economic status* | *Ethnicity-deprivation index* | *Age* | *Gender* |  |  |  |
| Countryside visits | -0.14  (-0.24 to -0.03) |  | 0.55  (0.34 to 0.76) | -0.33  (-0.56 to -0.09) | 0.00  (-0.01 to 0.01) | 0.35  (-0.05 to 0.75) | 1133.61 | 0 | 0.61 |
| " | -0.13  (-0.24 to -0.03) | 0.14  (-0.30 to 0.58) | 0.54  (0.33 to 0.75) | -0.32  (-0.56 to -0.08) | 0.00  (-0.01 to 0.01) | 0.35  (-0.05 to 0.75) | 1135.35 | 1.74 | 0.25 |
| " |  |  | 0.59  (0.37 to 0.80) | -0.45  (-0.67 to -0.23) | 0.00  (-0.01 to 0.02) | 0.34  (-0.07 to 0.74) | 1137.52 | 3.91 | 0.09 |
| Urban green-space visits |  | -0.54  (-0.99 to -0.08) | 0.76  (0.54 to 0.99) | -0.39  (-0.62 to -0.15) | 0.00  (-0.01 to 0.02) | 0.26  (-0.16 to 0.69) | 1169.63 | 0 | 0.57 |
| " | -0.03  (-0.14 to 0.09) | -0.55  (-1.02 to -0.09) | 0.76  (0.53 to 0.98) | -0.37  (-0.62 to -0.11) | 0.00  (-0.01 to 0.02) | 0.26  (-0.16 to 0.69) | 1171.57 | 1.94 | 0.22 |
| " |  |  | 0.72  (0.49 to 0.95) | -0.32  (-0.56 to -0.08) | 0.00  (-0.01 to 0.01) | 0.26  (-0.17 to 0.69) | 1172.23 | 2.59 | 0.16 |
| Biodiversity knowledge |  |  | 0.27  (0.17 to 0.37) | -0.23  (-0.34 to -0.12) | 0.02  (0.01 to 0.02) | 0.01  (-0.18 to 0.20) | 726.15 | 0 | 0.21 |
| Behavioural conservation support |  | -0.28  (-0.51 to -0.05) | 0.22  (0.11 to 0.34) | 0.08  (-0.04 to 0.20) | 0.00  (-0.01 to 0.00) | -0.16  (-0.38 to 0.05) | 782.01 | 0 | 0.60 |
| " | 0.01  (-0.05 to 0.07) | -0.27  (-0.51 to -0.03) | 0.23  (0.11 to 0.34) | 0.07  (-0.06 to 0.20) | 0.00  (-0.01 to 0.00) | -0.17  (-0.38 to 0.05) | 783.93 | 1.9 | 0.23 |
| " |  |  | 0.21  (0.09 to 0.32) | 0.11  (-0.01 to 0.23) | 0.00  (-0.01 to 0.00) | -0.17  (-0.39 to 0.05) | 785.31 | 3.30 | 0.11 |
| Financial conservation support | -0.05  (-0.10 to 0.00) |  | 0.20  (0.10 to 0.31) | -0.07  (-0.19 to 0.05) | 0.00  (-0.01 to 0.00) | -0.13  (-0.31 to 0.06) | 713.67 | 0 | 0.49 |
| " |  |  | 0.22  (0.12 to 0.32) | -0.11  (-0.22 to -0.01) | 0.00  (-0.01 to 0.01) | -0.13  (-0.33 to 0.06) | 715.01 | 1.34 | 0.25 |
