# Supplementary material for: Contrasting effects of visiting urban green-space and the countryside on biodiversity knowledge and conservation support
Source: PLoS One. 2017 Mar 23;12(3):e0174376. doi: 10.1371/journal.pone.0174376 (PMC5363982; doi:10.1371/journal.pone.0174376)
Supplement: S1 File — (PDF) [file pone.0174376.s014.pdf]

Questionnaire

1. How long have you lived in your current home? ______ *months or* ______ *years*

2a. How much built up land (i.e. buildings, roads, carparks etc) is there within a 5 minute walk from:

	None	Very little	A fair amount	A lot	Completely surrounded
i) where you currently live	□	□	□	□	□

2b. How much greenery is there within a 5 minute walk from:

	None	Very little	A fair amount	A lot	Completely surrounded
i) where you currently live	□	□	□	□	□

3. What is your current home postcode?___________________

4. If you had £600 to spend on charities from the following sectors, how much (if any) would you give to each type of charity?

a) Medical research £________
b) Animal and plant conservation £________
c) Environmental organisations £________
d) Human rights £________
e) Animal welfare/protection £________
f) Helping vulnerable people £________ Total______

5. On average over the course of a year, how often do you visit urban green-spaces (exc your garden)?
Daily □ Several times a week □ Once a week □ Fortnightly □ Monthly □ Once every 2 or 3 months □ Once/twice a year □ Never □

6. On average over the course of a year, how often do you visit the countryside?
Daily □ Several times a week □ Once a Week □ Fortnightly □ Monthly □ Once every 2 or 3 months □ Once/twice a year □ Never □

7. On average over the course of a year, how often do you spend time in your garden?
No garden □ Daily □ Several times a week □ Once a week □ Fortnightly □ Monthly □ Once every 2 or 3 months □ Once/twice a year □ Never □

8. On average over the course of a year, how often do you watch or listen to natural history programmes i.e. wildlife/nature documentaries?
Daily □ Several times a week □ Once a week □ Fortnightly □ Monthly □ Once every 2 or 3 months □ Once/twice a year □ Never □

9. Are you a current member of any charities (e.g. Amnesty International, English Heritage, Greenpeace, National Trust, RSPB?) Yes □ No □
If yes, which ones____

10a. Have you given any donations to other charities over the past month (either standing orders or one off donations)? Yes □ No □

10b. If yes, which ones?
____

10c. And how much to each per month? *Draw arrows*

£1-£5   £6-£10   £11-£20   £21-£40   £41-£60   £61-80   £81-100   More than £100

Or one off donation *how much was this if you're happy to tell us* £______________________________

11a. Do you currently volunteer for any groups? Yes □ No □

11b. If yes, which ones? ____

11c. And how frequently?____

12. Using scale A on your answer card, could you please tell me how strongly the following statements match your opinion? *Strongly agree, Agree, Neither agree/disagree, Disagree, Strongly disagree*

a) It takes too much time and effort to do things that are environmental friendly _____

b) Scientists will find a solution to global warming without people having to make big changes to their lifestyle____

c) The environment is a low priority for me compared with a lot of other things in my life _____

13.Can you please take a look at these three sets of photos and for each set rank them in terms of their value for wildlife so for example A is best, then B , then C or A and B are joint best then C.

a) Woodland Order (best first) __________

b) Farmland Order (best first) __________

c) Wetland Order (best first) __________

14. Please can you tell me if you can name any of these species and which ones are of conservation concern, i.e. have suffered major declines in numbers or range in the UK

	Name	Of Conservation Concern?		
		Yes	No	Really No idea
**Bird a**				
Bird b				
Bird c				
Bird d				
**Mammal a**				
Mammal b				
Mammal c				
Mammal d				
**Plant a**				
Plant b				
Plant c				
Plant d				

15. Could you please use scale B) on your answer card and tell us on a scale of 0 to 8 scale, how strongly the following statements match your opinion. *0=Do not agree at all, 4=Agree somewhat, 8=Agree completely*

a) I am willing to give things up that I like doing if they harm the natural environment _____

b) I am willing to take on responsibilities that will help conserve the natural environment _____

c) I am willing to do things for the environment, even if I'm not thanked for my efforts _____

d) Even when it is inconvenient to me, I am willing to do what I think is best for the environment _____

e) I am willing to go out of my way to do what is best for the environment _____

16. Which of the following categories apply to you?
Retired □ Unemployed □ Full-time education □ Full-time paid employment □
Part-time paid employment □ Self-employed □ Home maker/bringing up family □

17. What's the highest level qualification that you have?

None □ O level/GCSE or equivalent □ A level or equivalent □ Undergraduate degree □ Higher degree □
Vocational qualification (state level if known____) □

18. What tax band are you in?

No tax (<£9,440 taxable income) □ Basic rate (£9,440-£32,010) □

Higher rate (£32,011-£150,000) □ Top rate (£>150,000) □

19. Which age category are you in?

16-18 □ 19-24 □ 25-34 □ 35-44 □ 45-54 □ 55-64 □ 65-74 □ 75-84 □ 85-94 □ 94+ □

20. Please take a look at this card and choose one option that best describes your ethnic group or background

____

Respondent's gender Male □ Female □

White
1. English / Welsh / Scottish / Northern Irish / British
2. Irish
3. Gypsy or Irish Traveller
4. Any other White background, please describe

Mixed / Multiple ethnic groups
5. White and Black Caribbean
6. White and Black African
7. White and Asian
8. Any other Mixed / Multiple ethnic background, please describe

Asian / Asian British
9. Indian
10. Pakistani
11. Bangladeshi
12. Chinese
13. Any other Asian background, please describe

Black / African / Caribbean / Black British
14. African
15. Caribbean
16. Any other Black / African / Caribbean background, please describe

Other ethnic group
17. Arab
18. Latin American
19. Any other ethnic group, please describe
